# The effect of anxiety on brain activation patterns in response to inspiratory occlusions: an fMRI study

**DOI:** 10.1038/s41598-019-51396-2

**Published:** 2019-10-21

**Authors:** Pei-Ying S. Chan, Yu-Ting Wu, Ai-Ling Hsu, Chia-Wei Li, Changwei W. Wu, Andreas von Leupoldt, Shih-Chieh Hsu

**Affiliations:** 1 Department of Occupational Therapy and Healthy Aging Center, Chang Gung University, Taoyuan, Taiwan; 2 Department of Psychiatry, Chang Gung Memorial Hospital at Linkou, Taoyuan, Taiwan; 3 Department of Radiology, Wan Fang Hospital, Taipei Medical University, Taipei, Taiwan; 4 Brain and Consciousness Research Center, Shuang-Ho Hospital, New Taipei, Taiwan; 5 Graduate Institute of Mind, Brain and Consciousness, Taipei Medical University, Taipei, Taiwan; 6 Health Psychology, University of Leuven, Leuven, Belgium

**Keywords:** Respiration, Cortex

## Abstract

Respiratory sensations such as breathlessness are prevalent in many diseases and are amplified by increased levels of anxiety. Cortical activation in response to inspiratory occlusions in high- and low-anxious individuals was found different in previous studies using the respiratory-related evoked potential method. However, specific brain areas showed different activation patterns remained unknown in these studies. Therefore, the purpose of this study was to compare cortical and subcortical neural substrates of respiratory sensation in response to inspiratory mechanical occlusion stimuli between high- and low-anxious individuals using functional magnetic resonance imaging (fMRI). In addition, associations between brain activation patterns and levels of anxiety, and breathlessness were examined. Thirty-four (17 high- and 17 low-anxious) healthy non-smoking adults with normal lung function completed questionnaires on anxiety (State Trait Anxiety Inventory - State), and participated in a transient inspiratory occlusion fMRI experiment. The participants breathed with a customized face-mask while respiration was repeatedly interrupted by a transient inspiratory occlusion of 150-msec, delivered every 2 to 4 breaths. Breathlessness was assessed by self-report. At least 32 occluded breaths were collected for data analysis. The results showed that compared to the low-anxious group, the high-anxious individuals demonstrated significantly greater neural activations in the hippocampus, insula, and middle cingulate gyrus in response to inspiratory occlusions. Moreover, a significant relationship was found between anxiety levels and activations of the right inferior parietal gyrus, and the right precuneus. Additionally, breathlessness levels were significantly associated with activations of the bilateral thalamus, bilateral insula and bilateral cingulate gyrus. The above evidences support stronger recruitment of emotion-related cortical and subcortical brain areas in higher anxious individuals, and thus these areas play an important role in respiratory mechanosensation mediated by anxiety.

## Introduction

Respiratory sensations such as breathlessness are aversive and prevalent symptoms in many cardiorespiratory diseases. Brain mechanisms underlying respiratory mechanosensation remain poorly understood, thus, limiting our understanding of mechanisms that contribute to the experience of breathlessness. In the past two decades, respiratory-related evoked potential (RREP) studies have been conducted, providing excellent temporal resolution for investigating the neural processing of respiratory sensations such as respiratory occlusions by electroencephalography^1–6^. Short- and long- latency RREP peaks are indicative of the exogenous and endogenous nature of respiratory sensory processing of mechanical stimuli^7–9^. Source analyses and animal studies suggested that respiratory occlusions elicited activations over several brain areas including the somatosensory cortex (SI), somatosensory association cortex (SII), prefrontal, premotor, and supplementary motor cortices as well as over parietal areas, thalamus, and brainstems^8,10–13^. However, the limited spatial resolution of the RREP technique prevents robust interferences about the specific localisations of these activations within the brain. Therefore, recent studies have been using the high spatial resolution of the functional magnetic resonance imaging (fMRI) technique to investigate cortical and subcortical areas related to respiratory mechanosensation^14–23^. Most studies have utilized resistive loads to measure brain substrates related to dyspnea^14,15,17,19,22,24,25^, whereas a few other studies have used inhalation of elevated CO_2_ levels^26,27^ or threshold loads, respectively^23^. These studies found that not only sensorimotor areas such as the sensorimotor cortex, supplementary motor area, SII and thalamus, but also emotion-related areas such as insular cortex, amygdala, anterior cingulate cortex (ACC), hippocampus, and periaqueductal gray (PAG) were activated with inspiratory resistive loads. Partly, these activations in emotion-related areas such as insula, ACC, hippocampus and amygdala were associated with elevated levels of negative affect such as anxiety or increased unpleasantness of breathlessness^14,15,19,21,25,28–31^. In addition, a few researchers have used transient inspiratory occlusion in a single breath to measure brain activation associated with breathlessness^16,18^. For example, Jack *et al*. (2010) suggested that transient inspiratory occlusions (TIO) elicited activation in sensorimotor, pre-motor, and insular cortices in patients with idiopathic hyperventilation^16^. In our recent study, we found that TIO elicited significant brain activations in the thalamus, caudate, premotor area, and SII^18^.

Whether these brain activation patterns in response to transient inspiratory occlusions would differ between low- and high- anxious individuals remained unclear from these studies. However, various behavioural studies have provided evidence that anxiety is associated with elevated levels of breathlessness^29,32–36^. For example, greater anxiety was found associated with elevated levels of dyspnea during ergometer exercise^32^ and during activities of daily life in patients with COPD^37^. Marines-Price and the colleagues (2019) found that levels of dyspnea on exertion is associated with subjective ratings of anxiety and negative emotions in healthy obese women^36^. Moreover, RREP studies demonstrated that in an aversive environment, anxious individuals directed more neural processing capacities to respiratory sensory stimuli compared to a neutral environment^38^. Similarly, high-anxious individuals were also found to show decreased central neural inhibitory function in respiratory sensation^39–42^. However, the specific cortical and subcortical brain areas underlying these different activation patterns to TIO in individuals with different levels of anxiety were not identified.

Therefore, the purpose of this fMRI study was to identify differences between high- and low-anxious individuals in their cortical and subcortical brain activation patterns associated with respiratory mechanosensation as elicited by inspiratory occlusions. Across all individuals, we expected to observe neural activation in the somatosensory cortex, middle frontal cortex, the inferior parietal cortex, lentiform nucleus, caudate, and thalamus, as in our earlier study using TIO^18^. More specifically, we further hypothesized that high compared to low anxious individuals would demonstrate different levels of brain activations in emotion-related brain areas including the insula, hippocampus, and cingulate cortex. In additional explorative analyses we examined the associations between brain activation patterns and individual levels of anxiety and breathlessness.

## Methods

### Participants

Healthy nonsmoking adults with self-reported absence of cardio-respiratory and/or neurological diseases were pre-screened to ensure there were no metal implants, pacemakers or braces, and claustrophobia. Women with an age over 50 years were excluded to avoid potential confounding effects of menopause-related symptoms. A total of 34 participants (20 females, mean age = 23 ± 3.4 years) signed the informed consent and participated in the study. This study was reviewed and approved by the Institutional Review Board of the Chang Gung Medical Foundation (106-0880D). All methods in this study were performed in accordance with the relevant guidelines and regulations.

### Respiratory apparatus

The general setting of the apparatus was described previously^18,43–45^. MRI experiments were conducted by a 3T MRI scanner (Prisma, Siemens, Erlangen, Germany) with 20-ch head coil at National Taiwan University. Briefly, participants were laying in supine position in the MRI scanner. They were breathing through a two-way non-rebreathing valve with a facemask (Hans Rudolph Inc., Kansas City, USA). The participant’s mouth pressure was monitored by a pressure tubing at the center of the non-rebreathing valve connected to a differential pressure transducer of a pneumotachograph amplifier (1110 series, Hans Rudolph Inc., Kansas City, MO, USA) and a PowerLab signal recording unit (ADInstruments Inc., Bella Vista, NSW, Australia). The two-way non-rebreathing valve was connected to a customized occlusion valve (Hans Rudolph Inc., Kansas City, USA), which can be controlled manually. To be compatible with the MRI requirement, the customized occlusion valve was placed 3 meters away from the MRI scanner (Hans Rudolph Inc., Kansas City, USA). The occlusion valve was manually controlled by the experimenter via a customized trigger outside of the scanning room. For the stimulus events, the experimenter manually triggered a Transistor-Transistor Logic (TTL) pulse to activate the solenoid, which allows the pressure air tank to close the occlusion valve.

### Experiment protocol

After signing the consent form, participants performed a spirometric pulmonary function test to ensure that their baseline lung function (forced expiratory volume in 1 second, FEV_1_) was at least 70% of the normative values^46^. Then, the participants filled out the demographic information sheet and the State-Trait Anxiety Inventory (STAI) questionnaire. The STAI has established reliability and validity and is commonly used as a tool for measuring subjective ratings on state and trait levels of anxiety^47^. We used the state-version containing 20 items with the summary scores ranging from 20 (not anxious at all) to 80 (extremely anxious). Upon completion, participants were assisted to position the facemask and earplugs while laying supine with the head immobilized in the scanner head coil. The participants were instructed to breathe as normal as possible throughout the experiment. Occasional inspiratory occlusions of 150-ms were delivered randomly every 2 to 4 breaths at the onset of inspiration. The scan time was 12 minutes, which allowed at least 32 occluded breaths to be collected for data analysis. After the experiment, participants were given verbal instruction “please rate the level of breathless you experienced during this session that just ended” and rated their feeling of breathlessness during scanning using a Visual Analog Scale (0 = not at all breathless, and 100 = maximal level of breathlessness).

### Image acquisition

A T_1_-weighted image was acquired using a three-dimensional gradient-echo sequence (MP-RAGE) with an isotropic resolution of 1 mm. The fMRI experiment was performed using a 3-T whole-body scanner (Siemens MRI Scanner MAGNETOM Prisma, Erlangen, Germany) with a 20-channel head array coil. The blood oxygenation level-dependent (BOLD) response in fMRI signals was taken as the surrogate of local neuronal activation following each occlusion event. 32 continuous axial slices (thickness = 4 mm) were acquired by using a gradient echo, echo-planar T2*-sensitive imaging sequence (TR = 2 s, TE = 32 ms; flip angle = 90 degrees; iPAT factor = 2, matrix = 64 × 64; field of view = 220 × 220 mm^2^).

### Data analysis

The fMRI image processing and statistical analysis was performed with SPM8 (http://www.fil.ion.ucl.ac.uk/spm/, Department of Imaging Neuroscience, University College London, UK) and analysis of functional neuroimages (AFNI)^48^. All images were realigned to the first image, spatially normalized into standard anatomical space based on the MNI template and smoothed with an isotropic Gaussian kernel of 6-mm full-width at half-maximum. At the individual level, every short inspiratory occlusion was regarded as a stimulus event that induced corresponding hemodynamic responses, whereas the flat signal fluctuations during normal breaths were taken as the baseline. The statistical analysis was performed with the general linear model with time and dispersion derivatives, and the six motion parameters were included as nuisance regressors. After the model estimation, the generated beta map for every individual reflected the magnitude of the BOLD responses induced by transient inspiratory occlusions. At the group level, the activation map for all participants was determined using the voxel-wise one-sample *t*-tests with the significance level of p < 0.05 corrected for family-wise error (FWE) rate. Moreover, one-sample t-tests were used for the whole group of 34 participants to examine the group averaged beta values within selected regions of interest (ROIs) across the cortical and subcortical areas, where spherical ROIs were centered at the peak of activated brain areas with a radius of 5 mm.

Participants were then divided into a high anxious group (N = 17) and low anxious group (N = 17) based on a median split of the group’s STAI-S score (median = 36.5). Independent two-sample t-tests were used to examine the differences in brain activations between groups (high-anxious vs. low-anxious). To further investigate the group difference in brain activations, we reported the fMRI results at a corrected significance level of p < 0.05 using AFNI 3dClustSim correction concerning the autocorrelation function (uncorrected threshold of *p* < 10^−4^ and cluster size of 39 voxels). In addition, the ROI-based beta contrasts were used to examine the differences in BOLD activity between low- and high-anxious individuals.

Finally, associations of brain activation during inspiratory occlusions with individual levels of anxiety (STAI-S) and breathlessness (VAS) across the full group (N = 34) were examined by off-line correlation analyses (Pearson’s r) using SPSS software (SPSS Inc., Chicago, IL) using a significant level of p < 0.05.

## Results

**Figure 1:**
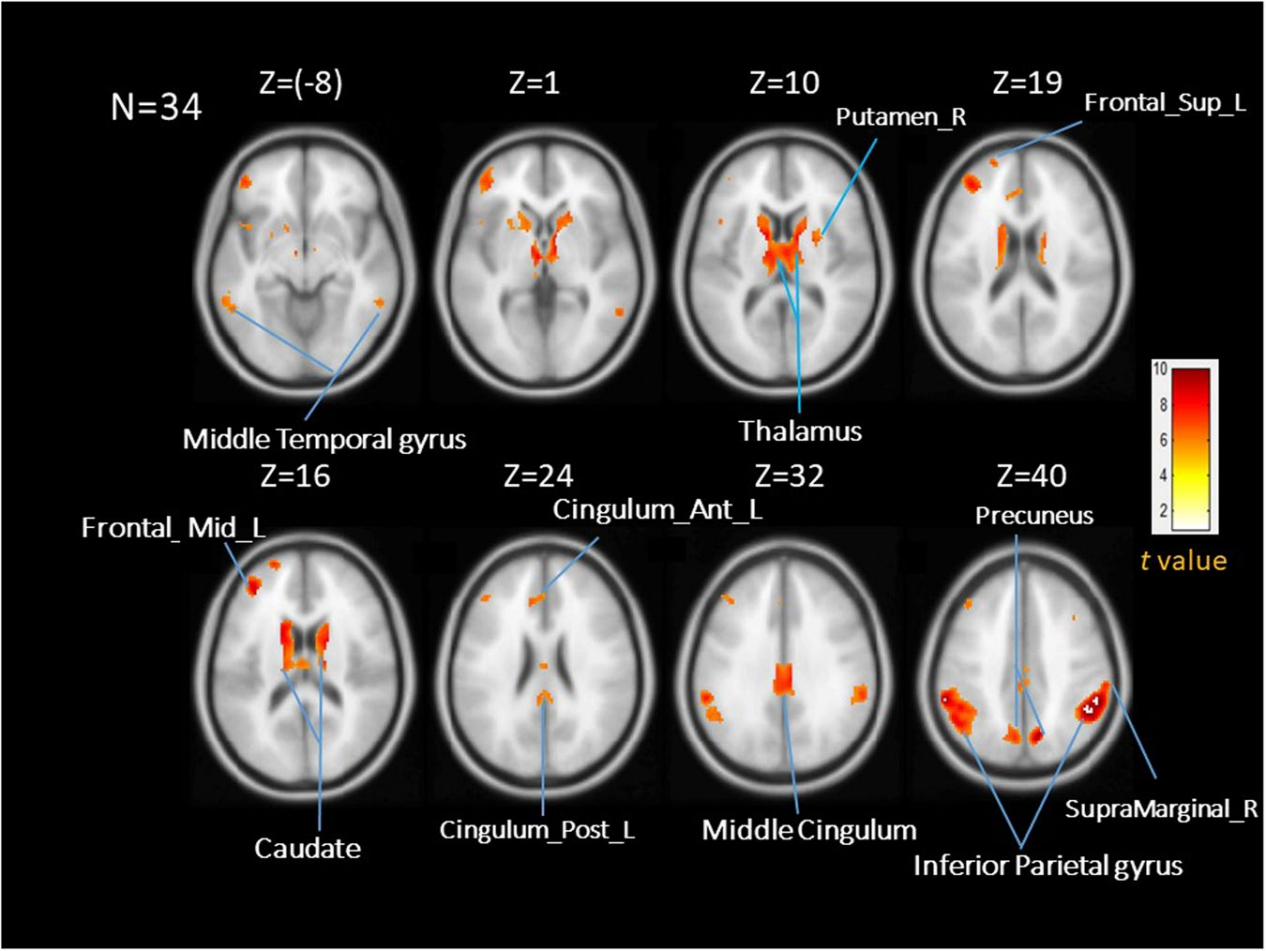
Averaged brain activation maps for the full group of participants (N = 34) during inspiratory occlusion compared to baseline conditions. Significant activations were observed in the thalamus, caudate, putamen, precuneus, supramarginal gyrus, cingulate cortex, temporal lobe, SII, frontal cortex, and inferior parietal cortex using a threshold of p < 0.05 (FWE corrected).

Table 1 presents baseline characteristics with mean (SD) for all participants. Briefly, 34 participants with 20 females and 14 males were tested. There was no significant difference between the high- and low-anxious groups in terms of age and FEV1 in % of predicted value. There were more females in the high-anxious group compared to the low-anxious group (13 and 7 females, respectively). As expected, the high-anxious group had higher average scores for the STAI, compared to the low-anxious group (44.76 ± 6.13 vs. 28.65 ± 4.9, p < 0.05). Figure 1 shows the activation map of the effect of inspiratory occlusions for the full group of 34 individuals. Significant activations were observed in the thalamus, caudate, putamen, precuneus, supramarginal gyrus, cingulate cortex, temporal lobe, SII, frontal cortex, and inferior parietal cortex using a threshold of p < 0.05 (FWE corrected).

**Table 1:** Baseline characteristics of the study participants (N = 34).

Variables	All subjects	LA	HA
N	34	17	17
Age (yrs)	23.4 ± 3.4	23.6 ± 4.4	23.1 ± 2.1
Gender (female/male)	20/14	7/10	13/4
FEV1 in % of predicted value	82 ± 8.3	84.6 ± 8.9	79.4 ± 6.8
FVC in % of predicted value	77.4 ± 9.8	81.2 ± 10.1	73.5 ± 7.7
STAI-S	36.7 ± 9.8	28.65 ± 4.90	44.76 ± 6.13*
Self-reported breathlessness (VAS)	32 ± 25	31.8 ± 25.3	32.2 ± 24.7

FEV1, forced expiratory volume in 1 sec; FVC, Forced vital capacity; STAI-S, The state-trait anxiety inventory, state scale; LA, lower anxious; HA, higher anxious. *Indicates a significant difference between the LA group and the HA group (p < 0.05).

**Figure 2:**
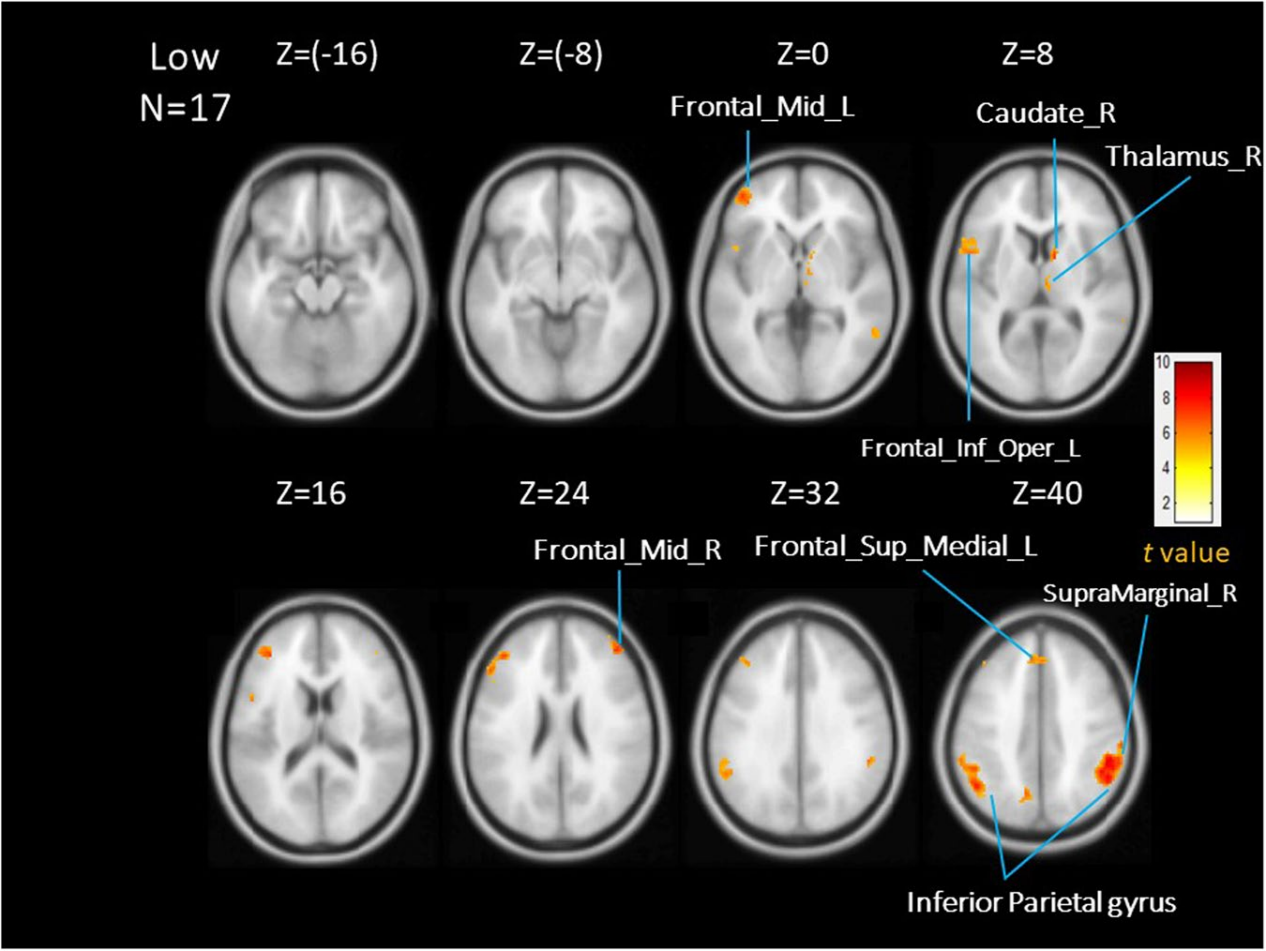
Averaged brain activations in the low-anxious group (AlphaSym corrected p < 0.05).

**Figure 3:**
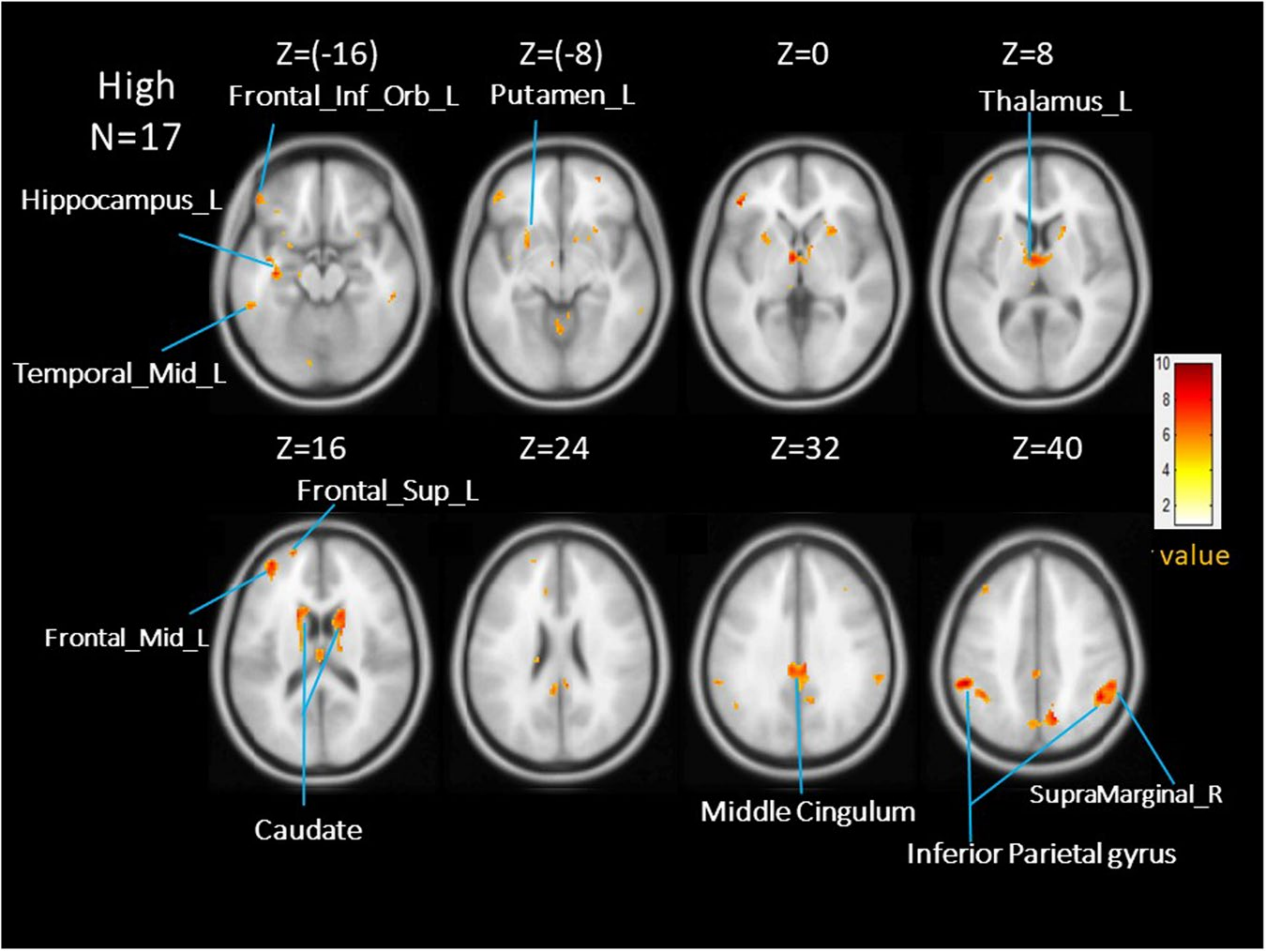
Averaged brain activations in the high-anxious group (AlphaSym corrected p < 0.05).

The subgroup analysis compared brain activations during inspiratory occlusions between the high- (N = 17) and low-anxious (N = 17) groups. The SPM analysis revealed no significant differences in activation under the FWE correction criteria between groups. However, the voxel-wise one sample t-tests revealed significantly activated areas within each group. Table 2 and Fig. 2 represent the regions significantly activated in the low-anxious group, whereas Table 3 and Fig. 3 represent the regions significantly activatied in the high-anxious group (corrected p < 0.05). Our results showed that low anxious individuals demonstrated higher levels of brain activation in the supramarginal gyrus compared to the higher anxious individuals. In contrast, the high-anxious group showed strong activations in the middle cingulate cortex, insular cortex, and hippocampus, which were not observed in the low-anxious group.

**Table 2:** Brain areas significantly activated in the low-anxious group (N = 17).

Region	R/L	X	Y	Z	Cluster	T value
Inferior parietal gyrus	R	54	−52	46	817	10.44
Supramarginal gyrus	R	54	−40	42		9.82
Middle frontal gyrus	L	−42	38	28	306	8.16
Middle frontal gyrus	R	44	44	22	88	8.05
Angular gyrus	L	−48	−58	40	724	7.87
Inferior parietal gyrus	L	−58	−42	48		7.28
Caudate	R	12	6	6	128	7.05
Thalamus	R	6	−18	4		6.07
Superior medial frontal gyrus	L	−6,	36	38	157	6.65
Middle temporal gyrus	R	64	−44	6	83	6.52
Precuneus	L	−10	−64	40	61	6.37
Superior parietal gyrus	L	−14	−72	42		5.37
pars opercularis of the inferior frontal gyrus	L	−50	6	18	145	6.22
Pars triangularis of the inferior frontal gyrus	L	−50	16	6		5.74

**Table 3:** Brain areas significantly activated in the high-anxious group (N = 17)

Region	R/L	X	Y	Z	Cluster	T value
Inferior parietal gyrus	L	−58	−38	38	393	9.28
Caudate	R	18	12	14	911	9.21
Thalamus	L	−2	−6	10		7.63
Middle Cingulum	L	−2	−32	44	331	8.81
Inferior parietal gyrus	R	48	−50	40	354	8.58
Supramarginal gyrus	R	56	−42	38		7.55
Middle Temporal gyrus	L	−62	−44	−12	78	8.04
Middle Frontal gyrus	L	−36	52	14	132	8.02
Hippocampus	L	−28	−16	−12	101	7.97
Insula	L	−38	−4	−12		7.02
Precuneus	R	14	−66	40	178	7.91
Pars triangularis of the inferior frontal gyrus	L	−44	36	0	130	7.89
Inferior orbital frontal gyrus	L	−46	42	−18		6.90
Superior frontal gyrus	L	−20	60	16	45	6.96
Putamen	L	−24	6	−8	85	6.19

**Figure 4:**
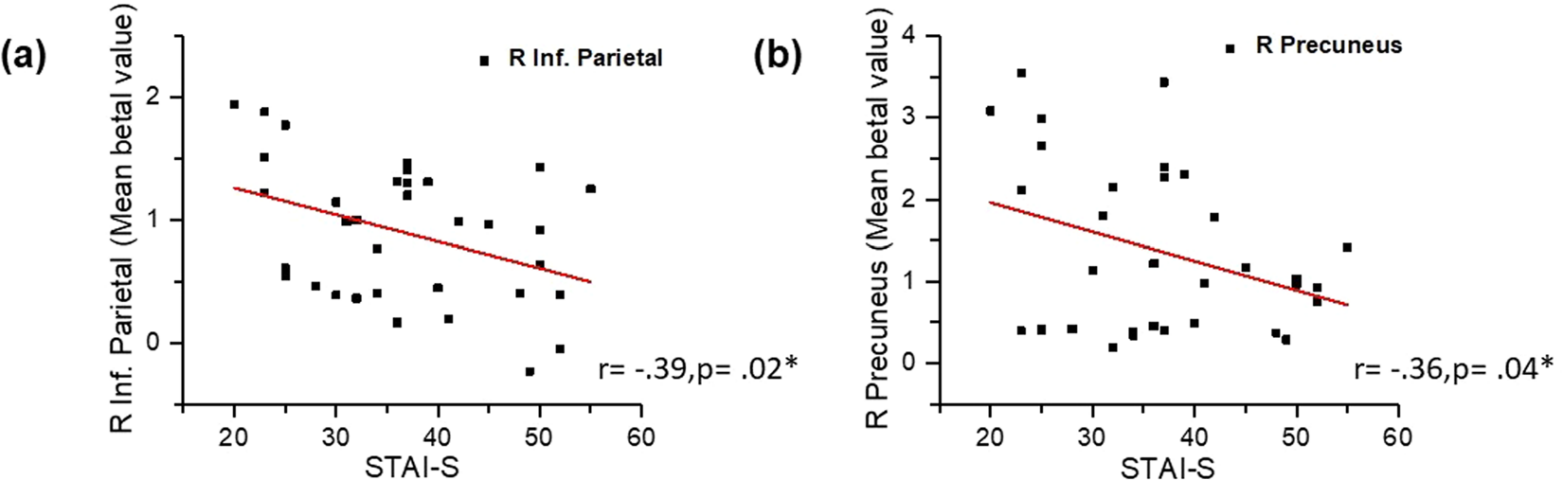
Scatter plots showing the correlations between the participants’ state anxiety levels and the brain activation levels (expressed as the beta values); Correlations between anxiety levels and activation in right inferior parietal cortex (**a**), and right precuneus (**b**).

**Figure 5:**
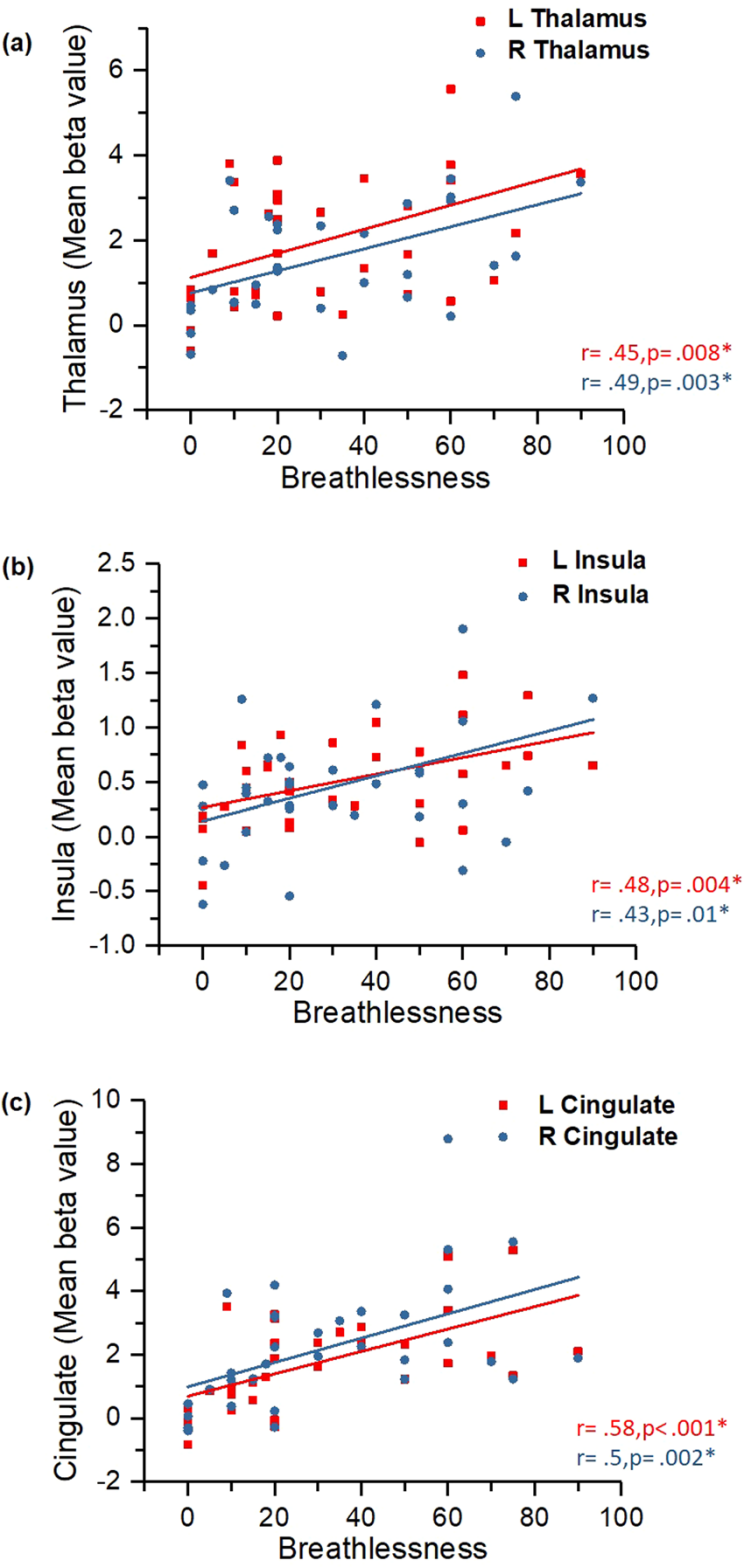
Scatter plots showing the correlations between the participants’ subjective ratings on breathlessness and the brain activation levels (expressed as the beta values); Correlations between breathlessness level and activation in bilateral thalamus (**a**), bilateral insula (**b**), and bilateral cingulate cortex (**c**).

Explorative correlational analyses showed that the STAI-S scores were negatively correlated with activation (beta values) of the right inferior parietal cortex (r = −0.39, p = 0.02), and the right precuneus (r = −0.36, p = 0.04) (see Fig. 4). In addition, levels of breathlessness were positively correlated with activation (beta values) of the right thalamus (r = 0.49, p = 0.003), the left thalamus (r = 0.45, p = 0.008), the right insula (r = 0.43, p = 0.01), the left insula (r = 0.48, p = 0.004), the right cingulate cortex (r = 0.50, p = 0.002), and the left cingulate cortex (r = 0.58, p < 0.001) (see Fig. 5).

## Discussion

The present study examined the brain activation patterns in response to transient inspiratory occlusions. We further analyzed the difference in the activated brain areas between a group of high- and low-anxious individuals and explored associations between levels of anxiety and breathlessness with brain activations. Our results demonstrated that cortical and subcortical regions including thalamus, inferior parietal lobe, frontal lobe, temporal lobe, and SII were activated by respiratory mechanical stimulation, consistent with our recent study^18^. Our results further revealed that low anxious individuals showed a significant level of brain activation, especially at supramarginal gyrus. The high-anxious group showed a strong activation level in the middle cingulate cortex, insular cortex, and hippocampus, which were not observed in the low-anxious group. Moreover, significant relationships were found between anxiety levels and activations of the inferior parietal gyrus and the precuneus as well as between breathlessness levels and activations of the thalamus, insula, superior frontal gyrus, and cingulate gyrus.

The present findings are consistent with earlier studies examining brain activations in response to respiratory stimulation and breathlessness^14,15,18–21,23,27^. For example, Evans *et al*.^27^ used hypercapnic breathing together with reduced tidal volume as manipulated by mechanical ventilation as experimental stimuli to test related brain substrates. Comparable to the present study, they found that thalamus, caudate, sensorimotor, parietal and prefrontal cortices, next to other areas, were related to dyspnea perception. McKay *et al*.^20^ investigated neural correlates of voluntary breathing and found that cortical and subcortical areas such as sensory motor areas, thalamus, and caudate were activated next to cerebellum and medulla. Raux *et al*.^23^ discovered that single threshold loaded breathing increased brain activations areas including premotor cortices, right parietal cortex, bilateral thalamus, and bilateral insula, whereas it decreased BOLD activities in other areas including the anterior cingulate cortices, and temporal cortices. Our earlier study in testing brain activations with similar mechanical stimuli also showed that thalamus, supramarginal gyrus, middle frontal cortex, and caudate were activated (Chan *et al*., 2018). Together, these studies suggest common activations in the thalamus, supramarginal gyrus, middle frontal gyrus, premotor cortices, parietal lobe, bilateral insula, and caudate during different forms of respiratory stimulation including inspiratory occlusions and loaded breathing.

Moreover, during the respiratory occlusion events, the low-anxious group showed a trend of more extensive hemodynamic response in the inferior parietal cortex (IPC), especially the supramarginal gyrus, and in the middle frontal gyrus, than the high-anxious group did. This trend suggests that the low-anxious group might have possessed more resources in subsequent neural processes after initial sensing of respiratory occlusions than the high-anxious group. This notion is paralleled by the findings in some previous reports in the field^38,42,45,49^. For example, Chan *et al*. (2014) found that patients with generalized anxiety disorder exhibited lower amplitudes of the RREP P3 peak, suggesting they used less neural resources to process attention-related tasks in respiratory sensation^45^. With high-density EEG methodology, von Leupoldt *et al*. (2011) further suggested that that the sources of P2 and P3 peaks of the RREP were likely diversified in the association cortices^38^. Miller & Cohen (2001) mentioned that the frontal area retains emotion-related memories from the limbic system^50^. Taken together, the above evidence indicates that long-latency RREP peaks were probably generated in both the secondary somatosensory cortices and association motor cortices, and higher-order cognitive processing of respiratory sensation is closely related to the individuals’ anxiety status^38,43,45^.

Our results also demonstrated that the high-anxious group had a trend of increased neural activation in some ROIs including the insula, middle cingulate cortex, and hippocampus during respiratory occlusion events, which were not observed in the lower anxious group. These findings are consistent with some previous studies examining the effects of anxiety and negative affect, or diseased states^14,15,19,21,25,29–31^. For example, Stoeckel *et al*. (2016) used resistive loading to elicit dyspnea sensation in healthy subjects. They found that the participants’ showed increased level of neural activations in the limbic system, where activation in the insula and anterior cingulate cortex (ACC) during anticipation were positively associated with anticipatory fear. This evidence together with another study of Stoeckel *et al*.’s (2018) indirectly supported our notion of state anxiety interacting with neural processing of respiratory mechanosensation. Moreover, in the study of Carlson *et al*.^51^ using an auditory anticipation task, the right insular was found predictive of the subjects’ anticipatory anxiety for aversive and neutral stimuli, whereas the amygdala was predictive of anxiety for aversive stimuli only. In our study, the transient inspiratory occlusions given intermittently during the inspiratory phase were not perceived as aversive stimuli by most subjects, which may be the reason that activation of the amygdala did not emerge to be significant in the current study^51^. In our study, the hippocampus was significantly activated in the higher anxious group. This is comparable with some previous studies where patients with anxiety were found to more easily overgeneralized for dangerous stimuli in different contexts^52,53^. It can be speculated that, in the higher-anxious group, our transient, non-threat occlusion stimuli still somehow mimic dyspneic experiences and triggered hippocampal activation, although this concept needs to be further investigated. Lastly, Esser *et al*.^17^ reported that emotion-related areas such as the hippocampus were more activated in patients with moderate-to-severe COPD compared to healthy controls during the anticipation of increasing dyspnea, which was related to increased anxiety levels in the patient group. The above evidence underlines the prominent role of the limbic system structures for mediating the effects of anxiety and negative affect on respiratory sensation.

The high- and low- anxious groups were divided based on the median of the participants’ anxiety score measured by the STAI. There was no significant difference between the two sub-groups on their reported breathlessness score measured by the VAS. The reason for this is that the VAS scale measures the subjects’ feeling of breathlessness, where the subjects may have mainly focused on rating the intensity of the breathlessness, rather than the aversive feeling, resulted from transient inspiratory occlusions. According to the literature, it is the affective aspect of the dyspnea that is directly related to the individuals’ emotional status^54^. Therefore, the difference in the reported breathlessness levels did not directly reflect the subjects’ anxiety levels in our study. Another possibility for the inconsistency between the anxiety levels and the dyspnea rating is that the study participants are all healthy adults with only high- and low- levels of anxiety states, rather than comparisons between healthy adults and patients with diagnosis, and therefore no differences were found statistically. Previous literature has demonstrated that negative affective states enhance patients’ dyspnea perception^55^. It will be interesting for future studies to test the brain activation patterns in response to respiratory occlusions in patients with the fMRI.

Our correlational analyses between the behavioural data and the brain activation levels showed several significant relationships. Although our participants did not rate the level of breathlessness intensity and level of breathlessness unpleasantness separately, our results showed that the higher the subjective ratings of overall breathlessness, the higher the activations in the thalamus, insula, and cingulate cortex. The relationship between the insula and the unpleasantness dimension of breathlessness was highlighted in some earlier studies^14,56^. In addition, our results demonstrated that the higher the anxiety of the individuals, the lower the activation of the right inferior parietal lobe and the right precuneus. This finding about the somatosensory association cortex again supports the previous findings of RREP studies where higher anxious individuals exhibited lower levels of neural activations in the cognitive processing of respiratory sensation^38,45^. In addition, Farb and colleagues (2013) also suggested that the precuneus is a part of the medial parietal network associated with respiratory interoceptive awareness^57^. In line with these previous reports, our results suggest that the precuneus might mediate respiratory interoception as a function of individuals’ emotional status.

In summary, the present study examined cortical and subcortical brain substrates in response to transient inspiratory occlusions in relation with the effect of individuals’ anxiety state. Our results suggest that, in response to transient inspiratory occlusions, higher anxious individuals have significant neural activations in the insular cortex, middle cingulate cortex, and hippocampus, which was not observed in lower anxious individuals. Also, individuals’ anxiety levels were closely associated with the neural activation levels in specific brain areas including the inferior parietal gyrus and precuneus, suggesting important roles of these areas in mediating cerebral respiratory sensation elicited by inspiratory occlusions.
